# Directional amplitude backscatter modulation with suppressed Doppler based on rotating resonant loop

**DOI:** 10.1038/s41598-022-26609-w

**Published:** 2022-12-20

**Authors:** Ashkan Azarfar, Nicolas Barbot, Etienne Perret

**Affiliations:** Grenoble INP, LCIS, University of Grenoble Alpes, 26000 Valence, France

**Keywords:** Engineering, Physics

## Abstract

The directional amplitude backscatter modulation with suppressed Doppler is demonstrated based on the scattering from a symmetrically rotating resonant loop. The concept is studied theoretically and experimentally with perfectly compatible results. The symmetrical rotation of the scatterer and the effect of radial resonance, as the two crucial points to realize the idea, are highlighted through the comparison between the symmetric and non-symmetric cases, and the results obtained for scatterers with and without radial resonance. The presented backscattering modulation technique provides an amplitude modulating waveform which is uniquely linked to the directional reradiation pattern of the rotating loop scatterer in a definite resonant mode. With the pure directional amplitude modulation (DAM) induced on the backscattered wave, the envelope waveform can be accurately retrieved form the received signal using the In-phase and Quadrature (IQ) representation. The contribution of the background in a real environment can be detected and removed to obtain the exact modulating waveform. This property of the proposed backscattering modulation method can be applied for sensing, localization, and identification purposes with high sensitivity, read range, and robustness.

## Introduction

Radio Frequency (RF) backscatter communication based on modulated reflectors has been firstly introduced in 1948^1^. Stockman^1^ has considered the concept with a quite general point of view in his work, and he has demonstrated that a modulated reflector can be realized based on any phenomenon which changes the scatterer properties so that, the backscattered wave is modified in terms of magnitude, phase, or polarization during the time. For a time-harmonic incident wave, any modification on these three backscattered wave parameters will be translated to an amplitude modulation, phase modulation, or a mixed amplitude-phase modulation on the received signal. From a structural view, the addressed modulation methods in Ref.^1^ can be classified to two main types which are applied for non-loaded scatterers (i.e. any scattering structure in which there is no loading point or port)^2^ and loaded scatterers^3^. The latter, called “variable-damping modulation” in^1^, is mainly realized by load-modulated antennas^4^, and it has been widely employed in classical UHF Radio Frequency IDentification (RFID)^5^ and Ambient Backscattering Communication (AmBC)^6^ where a modulating electronic component modifies the magnitude and phase^7^, or polarization^8,9^ of the reflection from the antenna during time. Usually, in this type, the energy required for the modulation is harvested from the incident electromagnetic wave, or the built-in battery, to power up the electronic component. On the other hand, in the former type based on moving non-loaded scatterers, which is more compatible with chipless RFID technology^10^, the required energy for modulation is provided by the motion source^1,11^.

In contrast to the loaded scatterer approach in which the data is stored in the electronic chip, for the non-loaded scatterer method the data can be associated either to the physical properties of the movement source (e.g. frequency and amplitude of the sound wave or periodic motion) or to the electromagnetic scattering properties of the scatterer (e.g. resonance frequency and reradiation pattern of the scatterer defined by its geometry). However, there are exceptional works like^12,13^ which cannot be fitted in this classification, since a load-modulated antenna has been used in Ref.^12,13^ while the modulation source is motion.

Following the first test examples demonstrated in Ref.^1^ with rotationally modulated scatterers (i.e. corner reflectors), rotational motion has been considered in wide variety of research works^14–26^ to utilize rotation induced backscattering modulation for sensing, classification, and identification goal. Most of the time, a rotating scatterer modifies a combination of the phase, magnitude and polarization on the backscattered wave, which yields a mixed amplitude-phase modulation on the received signal. In particular, the phase modulation caused by the radial velocity of the rotating scatterer towards the wave incidence direction (Doppler modulation), which is usually inevitable, has been extensively studied as micro-Doppler effect^27^. However, in most researches based on micro-Doppler effect^15,19–23^, the contribution of the phase modulation has not been clearly distinguished from the variation of the other wave parameters like magnitude and polarization, while they may present, and they have been considered jointly with the phase (micro-Doppler) modulation. In fact, since the dimension of the rotating scatterers studied in Refs.^19–23^ (respectively helicopter blades, wind turbine, corner reflector, star-shaped wheel, and Crookes radiometer) are much larger than the operating wavelength, they cannot be modeled as a point scatterer, and therefore they tends to induce a mixed amplitude-phase modulation on the received signal, even in a symmetrically rotating and non-depolarizing configuration. It should be mentioned that, this concept has been cleverly addressed far earlier by Ref.^17^ for scattering from jet engines in terms of received signal analysis, however, not in terms of scatterers behaviour. Taking into account the scatterer behaviour, the scatterer can be specially designed and configured to modulate only a specific wave parameter during the rotation to provide a pure amplitude or phase modulation. For example, rotating resonant dipole scatterers in Ref.^24^ have been configured such that the backscattered wave is modulated only in phase based on the micro-Doppler effect, which provides a pure analog phase modulation employed for RFID application. Moreover, to just modify the polarization of the reflected wave, dipole scatterers rotating perpendicularly respect to the incidence direction, which yields zero phase (Doppler) modulation, have been used in Ref.^26^ to achieve a pure amplitude modulation for sensing and identification.

According to the authors’ best knowledge, there is a lack of study to consider some special rotating scatterers which modulate the backscattered field only in magnitude, without affecting the phase^15,19–24^ or the polarization^25,26^ of the wave. “Directional scatterers” with definite reradiation pattern can be used to modify only the magnitude of the backscattered wave during the rotation, provided that the Doppler modulation is suppressed somehow. In other words, such kind of scatterer modulates the magnitude of the backscattered field during rotation with a waveform linked to the reradiation pattern of the scatterer. Worth mentioning that, although Stockman has addressed the DAM induced by “angular displacement” in his paper, neither him nor afterwards research works have discussed this concept analytically with a feasible example.

In this paper, we demonstrate that resonant scatterers, specifically the rectangular loop here, can provide a pure DAM when they are symmetrically rotated in a non-depolarizing configuration. As the key point, at resonant mode of the structure, the phase of the induced current on the scatterer is imposed by the mode, and the phase variation due to Doppler will be suppressed by the resonance effect, while the different points on the scatterer have a radial velocity towards the incidence direction. Moreover, the reradiation pattern of the scatterer is determined by the resonant current, and therefore, remains invariant during the rotation. This concept leads to a DAM on the backscattered wave, while the modulation waveform is uniquely defined by the reradiation pattern of the scatterer at the resonant mode.

**Figure 1:**
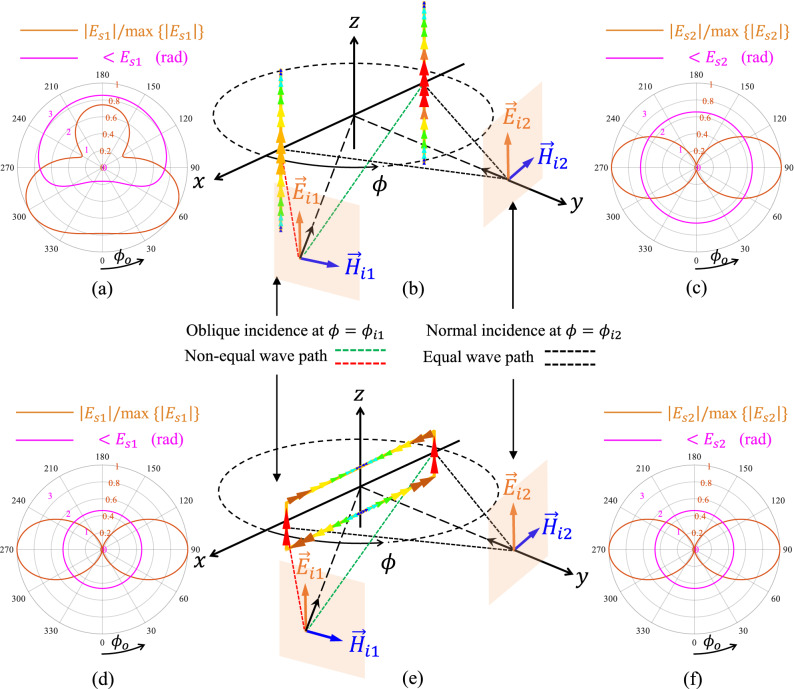
The azimuth distribution ($0^{\circ }<\phi _{o}<360^{\circ }$) of the phase and normalized magnitude of the scattered electric field from two dipoles for (**a**) oblique incidence (**c**) normal incidence. The azimuth distribution ($0^{\circ }<\phi _{o}<360^{\circ }$) of the phase and normalized magnitude of the scattered electric field from rectangular loop for (**d**) oblique incidence (**f**) normal incidence. Symmetrical and non-depolarizing scattering structure (**b**) without radial resonance [two equal-length coupled dipoles] (**e**) with radial resonance [rectangular loop]. All the data are obtained in simulation.

## Results

### Directional amplitude backscatter modulation

For a given scatterer impinged by a time-harmonic plane wave with defined polarization and incidence direction, the spatial distribution of the magnitude and phase of the scattered field components (the field components defines the polarization state of the scattered field) perfectly describes the modulation induced on the wave reflected from the rotating scatterer. Accordingly, for a definite rotation axis, to equivalently observe the variations during the rotation of the scatterer, the magnitude and phase variation of the scattered field components can be considered for different incidence angles corresponding to different rotation angles during the time.

The considered configuration in this paper is presented by Fig. 1 where a *z*-polarized plane wave impinges on the scatterers at different incidence angles $\phi =\phi _{i}$ (equivalent to rotation of the scatterers around *z*-axis), and the variation of the scattered field in terms of polarization, magnitude, and phase is investigated on *xy*-plane for observation angles $\phi =\phi _{o}$ ($0^{\circ }<\phi _{o}<360^{\circ }$). To preserve the polarization of the reflected wave during the rotation same as that of the incident wave, the scatterers should be designed in a non-depolarizing configuration such that the current path on them is fixed for all incidence angles, and the reradiated field has no cross-polarized component with respect to the incident wave. For the shown configuration in Fig. 1, vertically (*z*-) aligned plates (with relatively large dimensions compared to the wavelength), dipoles, and rectangular loops can provide a non-depolarizing condition. Moreover, in terms of phase variation, the geometrical symmetry of the scatterer about the rotation axis is a necessary condition leading to minimize the phase variation of the induced current on the scatterer for different incidence angles (and equivalently during the rotation of the scatterer). However, as the key contribution of the work, it will be shown that the geometrical symmetry and non-depolarizing configuration of a rotating scatterer are not sufficient conditions to modulate the backscattered wave only in magnitude, and the resonance effect should be included to suppress the phase variation due to Doppler.

To demonstrate the concept, two symmetric and non-depolarizing scattering configurations are considered as shown in Fig. 1b,e. The first one is composed of two equal-length *z*-aligned dipoles resonating in *z*-direction which are positioned symmetrically about the origin (Fig. 1b). The second one is a symmetric *z*-aligned rectangular loop resonating in *x*-direction (Fig. 1e). The resonance of the loop scatterer along *x*-direction (as shown in Fig. 1e) or more generally along the radial direction when the loop is rotated around *z*-axis is therefore called radial resonance since its direction is aligned with radial propagation of the incident wave in the proposed scattering configuration. Both structures are impinged at two different incidence angles ($\phi =\phi _{i1}\,,\,\phi _{i2}$), namely, oblique and normal incidence. For the oblique and normal incidence, the phase and magnitude distribution of the scattered field from the two dipoles are respectively shown in Fig. 1a,c, and those of the scattered field from the rectangular loop is respectively shown in Fig. 1d,f, while the scattered wave is co-polarized with the incident wave in the *xy*-plane for both structures. The magnitude of the scattered field have been normalized for all the cases to provide a better comparison between them. As it is illustrated in Fig. 1b, the phase of the induced current on the two dipoles depends on the wave path distances associated to each dipole (linked to the Doppler effect), which cause two dipoles to be in-phase for normal incidence (not shown in Fig. 1b) and to be out of phase for oblique incidence (shown in Fig. 1b). Consequently, the distribution of the phase and magnitude of the scattered field from two dipoles drastically changes as the incident angle varies (Fig. 1a,c). Nevertheless, for the rectangular loop with the radial resonance (resonating in *x*-direction), the phase of the induced current is imposed by the resonant mode, and it does not change for different incidence directions (for both normal and oblique incidence are the same, and it is shown in Fig. 1e) which means the Doppler effect has been suppressed due to the resonance. This phenomenon causes the azimuth distribution of the magnitude and phase of the scattered field to not vary for different incidence angles, while in addition, the phase distribution is constant as it is shown in Fig. 1d,f. Accordingly, this property can be used to realize a pure directional amplitude backscattering modulation. It should be mentioned that although the concept has been described for the fundamental resonant mode of the loop structure (the one shown in Fig. 1b), it is valid for all the resonant modes which produce a radial resonance.

**Figure 2:**
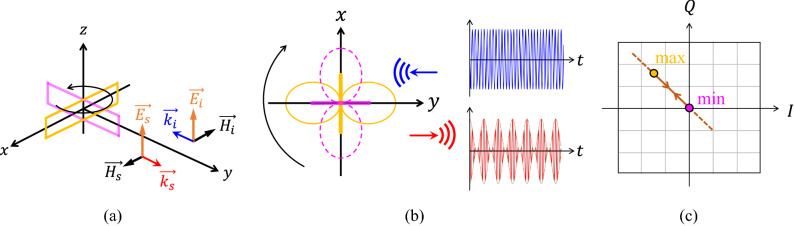
(**a**) Rotating rectangular loop in the monostatic scattering configuration. (**b**) Directional amplitude modulation induced on the backscattered wave by rotating loop. (**c**) The IQ constellation diagram of the demodulated received signal. The linear modulation path crosses the origin, and is limited in between the minimum and maximum of the reradiation pattern corresponding to the positions of the loop shown in (**b**).

The modulation process is presented in Fig. 2 for the monostatic scattering where $\vec {k}_{s}=-\vec {k}_{i}$ (Fig. 2a), and the rectangular loop is rotating around its symmetry axis (*z*-axis). As it is shown in Fig. 2b, the magnitude of the backscattered wave will be modulated during the rotation, while the envelope waveform of the received amplitude-modulated signal is exactly proportional to the directional reradiation pattern of the loop at the resonant mode. Applying a coherent IQ demodulation on the received signal yields the IQ constellation diagram presented in Fig. 2c. The modulation path is limited between two points corresponding to the maximum and minimum of the reradiation pattern, and coincided on a line which passes through the origin.

### Theoretical analysis

To verify the introduced concept, scattering from a rotating resonant rectangular loop is studied theoretically. A circuit model for the rectangular loop structure is proposed, and it is applied to develop an analytical model for the rotating loop. The model will be derived for general case (non-symmetrically rotating loop), and the effect of symmetry is highlighted for the special case (symmetrically rotating loop).

**Figure 3:**
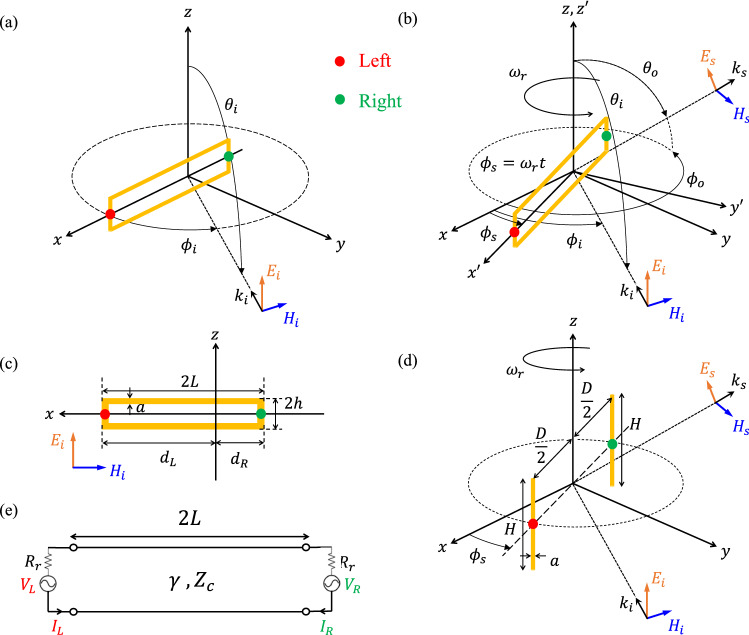
(**a**) Non-symmetrically positioned rectangular loop impinged by vertically polarized plane wave. (**b**) Scattering from non-symmetrically rotating rectangular loop. (**c**) Geometrical dimensions of the rectangular loop. (**d**) Scattering from two symmetrically rotating coupled dipoles. The left and right side radiating elements are respectively remarked with red and green dots. (**e**) The proposed circuit model for the wave-impinged loop.

#### Circuit model

Based on the induction theorem^28^, for a scatterer made of good conductors which is impinged by an electromagnetic wave, the induced current on the scatterer can perfectly describes the scattered field. Applying a circuit model to calculate the induced current on the scatterer (usually wire scatterers) is an efficient way to obtain the scattered field, provided that the circuit model is achievable. For a rectangular loop of wire, the circuit model is presented for the specific incident wave configuration shown in Fig. 3a. The rectangular loop with the length of 2*L* (in *x*-direction), the height of 2*h* (in *z*-direction), and wire radius of *a*/2 is non-symmetrically positioned in *xz*-plane such that $d_L$ and $d_R$ are respectively the distance between the left and right sides of the loop and the coordinate origin ($d_{L}+d_{R}=2L$) as it is depicted in Fig. 3c. The rectangular loop is impinged by a time-harmonic *z*-polarized plane wave at the frequency of $f_0$ with the wave vector of $\vec {k}_{i}=k\hat{k_{i}}$ ($\hat{k_{i}}$ is placed at *xy*-plane) where $k=2\pi f_{0}/c_{0}$ and $c_0$ is the free-space light velocity (Fig. 3a). For $h\ll L$ and $kh\ll 1$, it is shown in Ref.^29^ that the wave-impinged rectangular loop can be modeled by a section of two-wire transmission line with propagation constant of $\gamma \approx jk$ and characteristic impedance of $Z_{c}$, which is excited by two voltage generators at both left and right ends with respective complex ElectroMotive Force (EMF) of $V_L$ and $V_R$ (Fig. 3e). The voltage sources are followed with two identical resistors $R_{r}(\lambda )$ which models the radiation contribution of the two short end sides of the loop, while the negligible radiation of the transmission line section is ignored. In terms of reradiation, the two currents flowing on the left and right ends of the transmission line, namely $I_L$ and $I_R$, are the only current components which contribute in the scattered field. $I_L$ and $I_R$ can be calculated for given $V_L$ and $V_R$ based on the superposition principle and by using the even-odd mode decomposition technique (see Supplementary A). However, the EMF of the voltage sources ($V_L$ and $V_R$) should be obtained from the incident electric field, which leads to define a vector effective length associated to each resonant mode of the loop structure. Without lack of generality, and remarking that the model can be developed for all the even and odd resonant modes of the loop structure, at the rest of development we choose the dominant even mode of the rectangular loop as the case study (see Supplementary A).

The vector effective length of the loop at the fundamental resonant mode ($L=(c_0/f_0)/4=\lambda /4$) is derived based on the reciprocity theorem^28^ using the method proposed in Ref.^30^ (see Supplementary B). As the rectangular loop has been modeled by two reradiating current components on the left and right sides ($I_{L}=I_{R}=I_{0}$), there are two vector effective lengths ($\vec {l}_{e}^{\,L}$ and $\vec {l}_{e}^{\,R}$) obtained as

1 $$\begin{aligned} \vec {l}_{e}^{\,L}(\theta ,\phi )= & {} 2h\sin {\theta }\cos ({kL\sin {\theta }\cos {\phi }})\,e^{-jkL\sin {\theta }\cos {\phi }}\, {\hat{\theta }}, \end{aligned}$$

2 $$\begin{aligned} \vec {l}_{e}^{\,R}(\theta ,\phi )= & {} 2h\sin {\theta }\cos ({kL\sin {\theta }\cos {\phi }})\,e^{jkL\sin {\theta }\cos {\phi }} {\hat{\theta }},\end{aligned}$$

which are respectively associated to $I_{L}$ and $I_{R}$, and yield the induced EMF on each side in terms of the incident electric field as

3 $$\begin{aligned} V_{L}= & {} \vec {E}_{i}(r,\theta ,\phi )\,\cdot \, \vec {l}_{e}^{\,L}(\theta ,\phi )\,\Big |_{x=d_{L} \,,\, y=0\,,\,z=0}^{\theta =\theta _{i}\;,\;\phi =\phi _{i}}, \end{aligned}$$

4 $$\begin{aligned} V_{R}= & {} \vec {E}_{i}(r,\theta ,\phi )\,\cdot \, \vec {l}_{e}^{\,R}(\theta ,\phi )\,\Big |_{x=-d_{R} \,,\, y=0\,,\,z=0}^{\theta =\theta _{i}\;,\;\phi =\phi _{i}}, \end{aligned}$$

where $\theta _i$ and $\phi _i$ are determined by incidence direction (i.e. $\hat{k_i}$). As the induced EMFs are determined by (3) and (4) in terms of the incident field, the induced current on the loop and consequently the scattered field can be calculated using the proposed circuit model. The model will be applied to obtain the scattering from rotating loop in the next step.

#### Scattering from rotating rectangular loop

For a time-harmonic incident plane wave, scattering from a moving scatterer, when its velocity is much smaller than $c_0$, can be solved for time-dependent sequences of stationary scattered fields using the quasi-stationary approximation theory^31^. Thus, for the rotating loop, obtaining the current induced on the loop at each time instant during the rotation, while the loop is assumed fixed at the corresponding coordinate, makes it possible to derive the quasi-stationary scattered field.

Based on the developed circuit model, the induced current on the rotating loop can be obtained during the rotation by calculating the induced EMFs ($V_L$ and $V_R$) based on quasi-stationary approximation. Consider that the rectangular loop is non-symmetrically rotating around *z*-axis with angular rotation frequency of $\omega _r$ as it is shown in Fig. 3b, where the *xyz* is the reference Coordinate System (CS) and $x'y'z'$ is the local rotating CS attached to the rotating loop. The two coordinate systems are related by the rotation transformation with time-varying azimuth angle of $\phi _{s}(t)=\omega _{r}t=2\pi f_{r}t$ which can be expressed as

5 $$\begin{aligned} \begin{bmatrix} x \\ y \\ z \end{bmatrix} = \begin{bmatrix} \cos \phi _{s}(t) &{} -\sin \phi _{s}(t) &{} 0 \\ \sin \phi _{s}(t) &{} \cos \phi _{s}(t) &{} 0 \\ 0 &{} 0 &{} 1 \end{bmatrix} \begin{bmatrix} x' \\ y' \\ z' \end{bmatrix}. \end{aligned}$$

The electric field incident at the direction of $[\theta _{i}=\pi /2,\phi _{i}]$ can be written as

6 $$\begin{aligned} \vec {E_i}(\vec {r})=E_{0}e^{jk(x \cos {\phi _{i}} + y \sin {\phi _{i}})} \; {\hat{z}}, \end{aligned}$$

and the scattered field is observed at $[\theta _{o},\phi _{o}]$ direction. The induced EMFs during the rotation can be easily obtained by (3) and (4), provided that the incident field is expressed in the rotating CS. Using (5), the incident electric field can be written as

7 $$\begin{aligned} \vec {E_i}(\vec {r'})=E_{0}e^{jk[x' \cos {(\phi _{i}-\phi _{s})} + y' \sin {(\phi _{i}-\phi _{s})}]} \; {\hat{z}}, \end{aligned}$$

in the rotating CS. The time-varying complex envelope of the EMFs on the rotating loop (${\tilde{V}}_{L}(t)$ and ${\tilde{V}}_{R}(t)$) can be obtained using (1)–(4) and (7), while (1)–(4) should be rewritten in the rotating CS ($x'y'z'$) by replacing $r,\theta ,\phi$ with $r',\theta ',\phi '$. The resultant expressions for ${\tilde{V}}_{L}(t)$ and ${\tilde{V}}_{R}(t)$ are given by

8 $$\begin{aligned} {\tilde{V}}_{L}(t)= & {} \vec {E}_{i}(r',\theta ',\phi ')\,\cdot \, \vec {l}_{e}^{\,L}(\theta ',\phi ')\,\Big |_{x'=d_{L} \,,\, y'=0\,,\,z'=0}^{\theta '=\theta _{i}=\pi /2\;,\;\phi '=\phi _{i}-\phi _{s}}=2hE_{0}\cos {[kL\cos (\phi _{i}-\phi _{s})]}\; e^{jk\left(\frac{d_{L}-d_{R}}{2}\right)\cos (\phi _{i}-\phi _{s})}, \end{aligned}$$

9 $$\begin{aligned} {\tilde{V}}_{R}(t)= & {} \vec {E}_{i}(r',\theta ',\phi ')\,\cdot \, \vec {l}_{e}^{\,R}(\theta ',\phi ')\,\Big |_{x'=-d_{R} \,,\, y'=0\,,\,z'=0}^{\theta '=\theta _{i}=\pi /2\;,\;\phi '=\phi _{i}-\phi _{s}}=2hE_{0}\cos {[kL\cos (\phi _{i}-\phi _{s})]}\; e^{jk\left(\frac{d_{L}-d_{R}}{2}\right)\cos (\phi _{i}-\phi _{s})}, \end{aligned}$$

which are identical to each other, and yield in-phase and equal induced currents ${\tilde{I}}_{L}(t)={\tilde{V}}_{L}(t)/R_{r}$ and ${\tilde{I}}_{R}(t)={\tilde{V}}_{R}(t)/R_{r}$ on the rotating loop during the time. Therefore, the model is perfectly compatible with the proposed physical concept that the radial resonance of the rectangular loop will suppress the phase variation caused by the Doppler effect along the rotating loop.

The quasi-stationary scattered field form the rotating loop at the fundamental resonance can be calculated based on the currents (${\tilde{I}}_{L}(t)$ and ${\tilde{I}}_{R}(t)$) and by using the radiation integrals^28^. After some mathematical manipulations, the final expression for the time-varying complex envelope of the scattered field is obtained as

10 $$\begin{aligned} \vec {{\tilde{E}}}_{s}(\vec {r},t)&= {} \frac{j\eta k E_{0}(2h)^{2} \, e^{-jkr}}{2\pi r R_{r}}\; \sin {\theta _o}\cos {[kL\cos (\phi _{i}-\phi _{s})]} \;\cos {[kL\sin {\theta _o}\cos (\phi _{o}-\phi _{s})]}\nonumber \\{} & \quad \times e^{jk(\frac{d_{L}-d_{R}}{2})[\cos (\phi _{i}-\phi _{s})+\sin {\theta _o}\cos (\phi _{o}-\phi _{s})]}\; {\hat{\theta }}, \end{aligned}$$

where $\eta$ is the free-space intrinsic impedance and $\phi _{s}=\omega _{r}t$ is the time-varying rotation angle. The time-varying complex envelope can be rewritten as

11 $$\begin{aligned} \vec {{\tilde{E}}}_{s}(\vec {r},t)=\frac{j\eta k E_{0}(2h)^{2} \, e^{-jkr}}{2\pi r R_{r}}\; \sin {\theta _o}\; m(t) \;e^{j\psi (t)} \; {\hat{\theta }}, \end{aligned}$$

while *m*(*t*) and $\psi (t)$ are respectively associated to the DAM and the Doppler phase modulation, and can be described as

12 $$\begin{aligned} m(t)= & {} \cos {[kL\cos (\phi _{i}-\phi _{s})]} \;\cos {[kL\sin {\theta _o}\cos (\phi _{o}-\phi _{s})]}, \end{aligned}$$

13 $$\begin{aligned} \psi (t)= & {} k(\frac{d_{L}-d_{R}}{2})[\cos (\phi _{i}-\phi _{s})+\sin {\theta _o}\cos (\phi _{o}-\phi _{s})]. \end{aligned}$$

Obviously, for the symmetrical rotation ($d_{L}=d_{R}$), the Doppler modulation term will be completely cancelled $\psi (t)=0$ which provides a pure DAM with the waveform of *m*(*t*) linked to the reradiation pattern of the rectangular loop in its fundamental resonant mode. Thus, the proposed concept is proven theoretically by this demonstration.

### Simulation

The presented analytical model and the idea of the paper are verified by simulation. The two important effects to achieve a pure DAM, the symmetrical rotation and radial resonance of the scatterer, will be addressed well through the simulation results. For this, the two structures presented in Fig. 1 are considered. A rotating rectangular loop of wire with $L=15$ mm, $h=2$ mm, and $a=100~\upmu$m (Fig. 3b,d), and two rotating coupled dipoles with $H=30$ mm, $D=30$ mm, and $a=100~\upmu$m (Fig. 3e) are simulated in the monostatic scattering configuration with $\theta _{i}=\pi /2\,,\,\phi _{i}=\pi /2$ and $\theta _{o}=\pi /2\,,\,\phi _{o}=\pi /2$. The simulation process is described in “Methods”.

#### Symmetrical and non-symmetrical rotation

The symmetry of the scatterer about the rotation axis is the necessary condition to realize a pure DAM. In other words, even for the rectangular loop scatterer with radial resonance, it will be shown that the symmetrical rotation is essential for pure amplitude modulation, as it is predicted by the analytic model.

**Figure 4:**
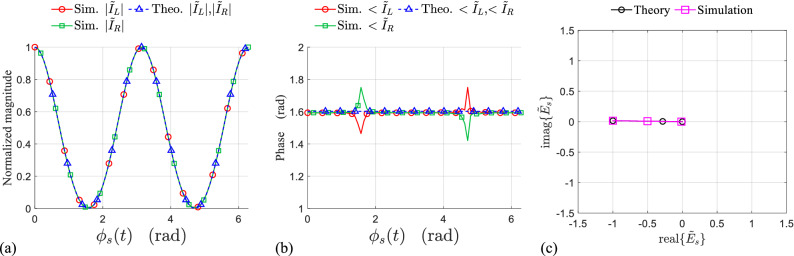
(**a**) Normalized magnitude, (**b**) phase of the induced currents on the left and right side of the symmetrically rotating rectangular loop during one period of rotation, obtained from the model and simulations. (**c**) IQ constellation of the time-varying complex envelope of the normalized backscattered field from symmetrically rotating loop in simulation and theory.

**Figure 5:**
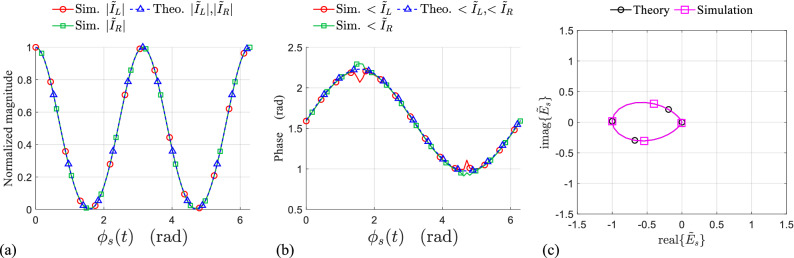
(**a**) Normalized magnitude, (**b**) phase of the induced currents on the left and right side of the non-symmetrically rotating rectangular loop during one period of rotation, obtained from the model and simulations. (**c**) IQ constellation of the time-varying complex envelope of the normalized backscattered field from non-symmetrically rotating loop in simulation and theory.

The phase and normalized magnitude of the induced currents on the left and right side of the rotating loop are extracted by simulation during one period of rotation $0<\phi _{s}<2\pi$. The simulation results for symmetrically ($d_L=d_R$) and non-symmetrically ($d_L\ne d_{R}$ , without lacking generality $d_L=3d_{R}$) rotating resonant loop at $f_0=5$ GHz are respectively shown in Figs. 4 and 5, both in perfect agreement with the results obtained from the model. Due to the radial resonance of the loop, for both cases (symmetrical and non-symmetrical rotation), the variation of the magnitude (Figs. 4a, 5a) and phase (Figs. 4b,  5b) of the left and right side currents [${\tilde{I}}_{L}(t)$ and ${\tilde{I}}_{R}(t)$] are identical to each other. However, the phase variation during the time is constant only for the symmetrical rotation (Fig. 4b), while it is varying sinusoidally for the non-symmetric rotation (Fig. 5b) resulting a phase modulation on the backscattered field. This fact has been clearly demonstrated in terms of IQ representation for the normalized backscattered electric field, and it is shown in Figs. 4c and 5c, where $I=real({\tilde{E}}_{s})$ and $Q=imag({\tilde{E}}_{s})$. As predicted by the model, the limiting points of the modulation path is determined by the maximum and minimum of the amplitude modulating waveform *m*(*t*) which is associated to the reradiation pattern of the resonant mode in the loop. Finally, it is worth-mentioning that the modulation path for the symmetric case ($d_L=d_R , \psi (t)=0$) is coincided on a line which pass through the coordinate origin (pure amplitude modulation), while that for the non-symmetric case ($d_L=3d_R , \psi (t)\ne 0$) follows a drop shape path as it is shown in Fig. 5c (mixed amplitude-phase modulation).

#### Symmetrical rotation: rectangular loop vs. two coupled dipoles

The importance of the radial resonance to achieve directional amplitude modulation based on rotating scatterer is clarified by simulation for two symmetrically rotating scatterers, namely the rectangular loop and two equal length coupled dipoles as it is depicted in Fig. 3d.

**Figure 6:**
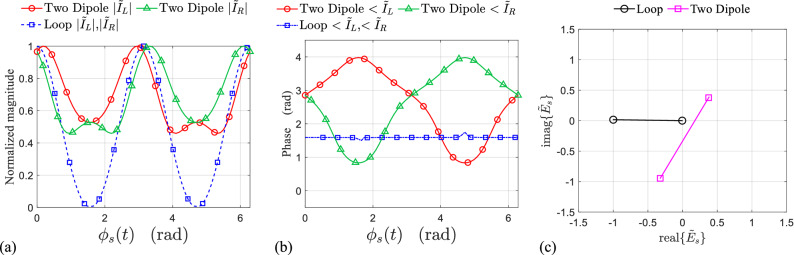
(**a**) Normalized magnitude, (**b**) phase of left-side and right-side induced currents on the two symmetrically rotating dipoles and the rectangular loop. (**c**) IQ constellation of the time-varying complex envelope of the normalized backscattered field from symmetrically rotating loop and two symmetrically rotating dipole. All the data are obtained in simulation.

For the rotating rectangular loop and two rotating dipoles, the simulation results are presented in Fig. 6a,b for the phase and normalized magnitude of the induced current on the left and right side elements during one period of rotation. Both simulations for the loop and the two dipoles have been done at $f_0=5$ GHz which is the fundamental resonance frequency of both scatterers. In contrast to the loop in which ${\tilde{I}}_{L}(t)={\tilde{I}}_{R}(t)$, the phase and magnitude variation of the induced currents on the left and right side dipoles are completely different. This fact, as it was discussed in Fig. 1, proves that, even in a symmetrical rotation, without the radial resonance, it is impossible to induce a pure DAM on the backscattered wave using a rotating scatterer. The IQ diagram of the normalized backscattered field shown in Fig. 6c also confirms that, although the modulation path for the two rotating dipoles is a line, however, it dose not pass through the origin, and consequently it represents a mixed amplitude-phase modulation.

### Experimental verification

At the final step, the proposed concept is validated through the measurement results. In this regard, the rectangular loop has been realized with a $250~\upmu$m radius copper wire, while the length and height of the loop are respectively $2L=29.8$ mm and $2h=3.9$ mm. The fundamental resonance of the loop has been measured at $f_0=4.98$ GHz, and the experimental study has been done using the monostatic setup which will be described in “Methods” section.

#### Environment contribution

Until this point, in the theoretical model and simulation, it has been assumed that the rotating loop is located in the free space. Nevertheless, to retrieve the same modulation waveform as the one obtained in theory from the measured backscattered signal, it is necessary to consider the contribution of the real environment in which the experiment has been done. Accordingly, it is supposed that in the measurement process the rotating loop is the only moving element in the environment, and all the other object in the environment are stationary. In addition, it is assumed that only the direct reflection of the modulated backscattered wave from the rotating loop is captured by the receiving antenna and the multi-reflection of the modulated wave with other present objects is much less significant compared to the direct reflection. With these assumptions, the backscattering contribution of the environment can be considered as a time-invariant term which is added to the complex envelope of the quasi-stationary backscattered field from the loop (10), and it is described as

14 $$\begin{aligned} \vec {{\tilde{E}}}_{s}^{meas}(\vec {r},t)=\vec {{\tilde{E}}}_{s}(\vec {r},t)+\vec {E}_{s}^{env}(\vec {r}), \end{aligned}$$

where $\vec {E}_{s}^{env}(\vec {r})$ will be observed as an added constant complex voltage $\rho _{env}$ in the demodulated received signal. Accordingly, in the IQ diagram representation, the contribution of the environment cause the modulation path to be translated along the constant vector of $\rho _{env}$ in the IQ plane. This is an important point for retrieving the directional amplitude modulation waveform associated to the reradiation pattern of the loop at its resonant modes. Actually, since for all the resonant modes of the loop (and of course for the fundamental mode) the reradiation pattern has some null points (zero magnitude as shown in Fig. 2b), the amplitude of the received signal will be more affected by noise at these points which makes the null points detectable on the modulation path in the IQ plane. Thus, the contribution of the environment ($\rho _{env}$) can be easily removed by putting the null point at the origin of the IQ plane, and then the modulation waveform is retrieved accurately. Note that this compensation cannot be done for the two symmetrically rotating dipoles since the modulation path does not cross the origin of the IQ plane. The measurement results in the next step will be processed based on this fact, and the DAM is validated experimentally.

#### Measurement results

**Figure 7:**
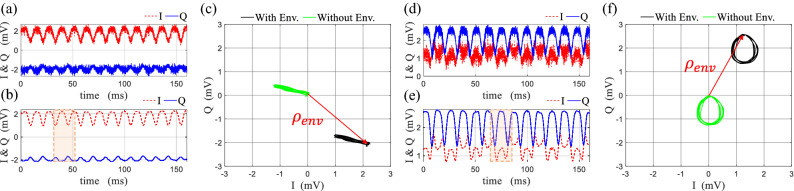
Obtained raw IQ data in measurement for scattering from (**a**) symmetrically, (**d**) non-symmetrically rotating rectangular loop, and the averaged IQ data for scattering from (**b**) symmetrically (**e**) non-symmetrically rotating rectangular loop. The measured IQ constellation diagram plotted based on the averaged data for (**c**) symmetrically (**f**) non-symmetrically rotating rectangular loop. The original measured modulation paths are shown in black color, and after the subtraction of the environment contribution (red arrow) they are shown in green color.

The measurements have been done for symmetrically and non-symmetrically rotating loop in time and frequency domain using the bench described in “Methods” section. After coherent IQ demodulation, the captured raw IQ data are presented for symmetrical and non-symmetrical rotation respectively in Fig. 7a,d. To represent the modulation path and to obtain the modulation waveform in a convenient form, the noise fluctuation on the raw IQ data have been reduced by applying moving average, and the resultant averaged data are presented in Fig. 7b,e for both cases. Based on the averaged IQ data, the modulation path is depicted for symmetrical and non-symmetrical rotation respectively in Fig. 7c,f [black curves], which are compatible with the predicted path in theory for both cases (line path for the symmetric rotation and drop shape path for the non-symmetric rotation).

**Figure 8:**
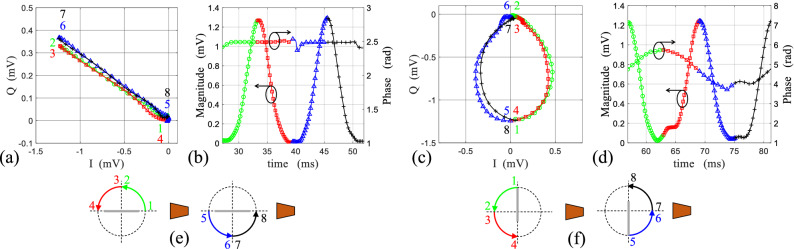
The modulation path during one period of rotation for (**a**) symmetrically, (**c**) non-symmetrically rotating loop. The magnitude and phase variation of the retrieved modulating waveform from measured signal for (**b**) symmetrically, (**d**) non-symmetrically rotating loop during one period of rotation. Each period of rotation is divided to four sections shown in green, red, blue and black color on the modulation path and modulating waveform. The respective orientation of the loop is given at each section for (**e**) symmetrical, (**f**) non-symmetrical rotation.

As it has been discussed previously, to retrieve the modulation waveform accurately, the contribution of the stationary environment $\rho _{env}$ should be removed from the data to achieve the original modulation path (Fig. 7c,f green curves). For each case, the original modulation path and the waveform are investigated on one period of rotation indicated in Fig. 7b,e by shadowed region ($27<t<52$ ms for symmetric rotation and $56<t<81$ ms for non-symmetric rotation), and are shown in Fig. 8. The modulation path can be divided to four quarter sections which correspond to the four quarter of the rotation with the respective orientation of the loop which is clearly illustrated by color markers in Fig. 8a,e for the symmetric case, and in Fig. 8c,f for the non-symmetric case. Moreover, these four sections are related to the modulation waveform in each case (Fig. 8b,d), while the magnitude variation is associated to the reradiation pattern of the fundamental resonance of the loop, and the phase variation is constant for the symmetric rotation (pure DAM with suppressed Doppler), and is sinusoidal for the non-symmetric rotation (mixed amplitude-phase modulation).

**Figure 9:**
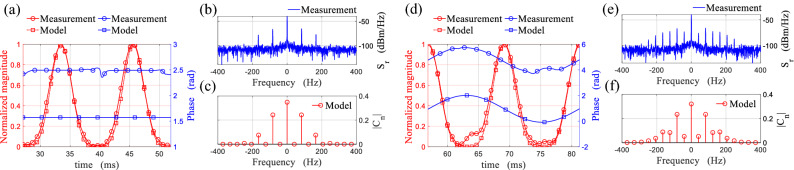
The magnitude and phase of the measured amplitude modulating waveform for (**a**) symmetrical, (**d**) non-symmetrical rotation of the loop compared with those predicted by the model. The measured PSD of the received signal backscattered from (**b**) symmetrically, (**e**) non-symmetrically rotating loop. The calculated Fourier coefficients of the time-varying complex envelope of the backscattered field from (**c**) symmetrically, (**f**) non-symmetrically rotating loop.

Finally, the normalized modulation waveform obtained from the measurement are compared with those predicted by the model in Fig. 9a,d, which shows a perfect agreement between the model and measurements. Furthermore, the Power Spectral Density (PSD) of the demodulated signal $S_r(f)$ has been measured for the symmetric and non-symmetric cases, and are respectively shown in Fig. 9b,e. The measured $S_r(f)$ can be compared with the calculated Fourier coefficients [$C_n$] of the backscattered field expressed in (10), which are presented in Fig. 9c,f. The measured and calculated frequency representation of the backscattered field contain components at the harmonics of the rotation frequency ($nf_r \; ; \;\; n=0,\pm 1, \pm 2, ...$). For the symmetric case, both in measurement and model, only the even harmonics exist (i.e. $2nf_r$), while for the non-symmetric case all the harmonics are present. Therefore, the frequency domain measurement results are also in accord with the proposed model in terms of harmonics and their respective ratios.

## Discussion

The backscatter modulation based on rotating resonant loop scatterer was proposed to introduce the first feasible example for DAM. The effect of radial resonance was utilized to suppress the phase modulation induced by Doppler which leads to a pure DAM on the backscattered wave. The concept was studied with an analytical model, and the model was verified by numerical simulations and measurements.

**Figure 10:**
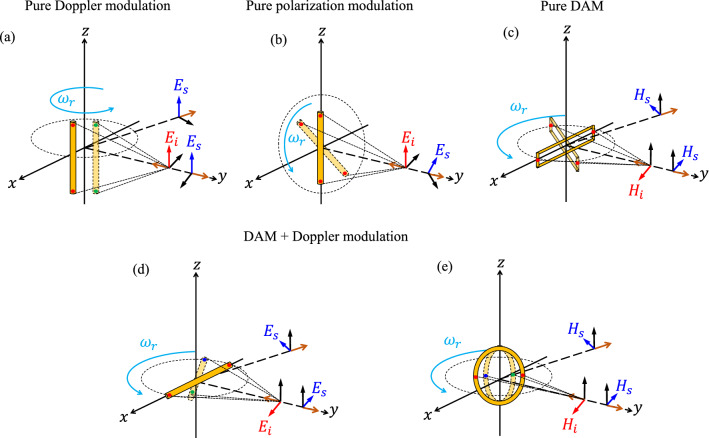
Backscatter communication based on rotating scatterers with (**a**) pure Doppler modulation, (**b**) pure polarization modulation, and (**c**) pure DAM. (**d,e**) backscatter communication with combined DAM and Doppler modulation. The phase change of the induced current on the scatterer during the rotation in (**d,e**) is shown by two red dots for normal incidence, and one blue and one green dot for oblique incidence.

Worth mentioning that, from the physical point of view, the proposed concept is quite specific since it clarifies that in a general backscatter modulation based on a moving scatterer, the modulation can be originated from three different physical phenomena as Doppler modulation, polarization modulation, and DAM, while the latter has not been clearly addressed before, and it has been usually merged with Doppler modulation. For rotating scatterers which have a radial velocity towards the incident wave, the Doppler modulation should be apparently involved. However, the suggestion of a backscatter modulation based on a rotating scatterer in which the modulation is done just proportionally to the directional property of the scatterer, perfectly clarifies the pure DAM and the Doppler suppression due to resonance effect, and separates its effect from the two other phenomena (Doppler and polarization modulation). To clarify this fact, Fig. 10a–c illustrates the three different rotation configurations which respectively provides a pure Doppler modulation^24^, pure polarization modulation^26^, and pure DAM (this work). Figure 10a presents the pure Doppler (phase) modulation in which the wire scatterer is aligned with the z-axis and is rotating around it. In this configuration, the backscattered field keeps the same polarization state as the incident field in all azimuth and elevation directions. Also, for when the radius of rotation is negligible compared to observation distance, since the reradiation pattern of the scatterer does not change during the rotation, there is no modulation in terms of magnitude (DAM) and polarization on the backscattered field. However, the phase (Doppler) modulation is shown in Fig. 10a by the phase change of the induced current during the rotation while the two different phases associated with two different radial distances are depicted by red and green color dots on the two endpoints of the scatterer. After, Fig. 10b shows the configuration for pure polarization modulation where the wire scatterer is lying in the xz-plane and is rotating around the y-axis and the incident wave impinges normally on the scatterer. In this case, the polarization state of the backscattered field is modulated during the rotation and the linear polarization state of the backscattered wave is rotating in its wave-front plane, while the phase and magnitude of the induced current on the scatterer and its reradiation pattern remain unchanged during the rotation. Since the radial distance of the scatterer respect to the incidence direction is fixed, the invariant phase of the induced currents at the two endpoints are depicted with red dots during the rotation. Finally, the resonant symmetrically rotating loop (proposed by this work) is shown in Fig. 10c where the backscattered wave keeps the same polarization state as the incident wave. The Doppler modulation due to the different radial distances is suppressed by the resonance effect and consequently, the reflected wave is modulated only due to the variation of the reradiation pattern of the scatterer during the rotation. In addition, to have some general examples (with the same considered scatterers) in which a combined modulation is involved, Fig. 10d,e presents two configurations (respectively horizontally rotating non-resonant dipole and symmetrically rotating non-resonant loop) while the backscattered wave experiences both Doppler modulation and DAM. Note that both configurations shown in Fig. 10d,e will also provide a pure DAM if the scatterer is resonant so that the Doppler modulation is suppressed.

Last but not least, since the pure DAM induced on the backscattered wave is proportional to the reradiation pattern of the resonant scatterer which usually has some null points, using coherent demodulation, the contribution of the environment can be easily removed to accurately retrieve the associated reradiation pattern from the received signal. This fact raises some potential applications for this kind of pure amplitude backscatter modulation such as pattern identification (where the reradiation pattern of the scatterer at each resonant mode can be assigned to a specific ID or different scatterers with specific patterns can be used for each ID like^22^), localization (where the general bistatic expression of the time-varying backscattered field (Eq. 10) is utilized to find the incidence $\phi _{i}$ and observation $\phi _{o}$ angles based on the retrieved amplitude profile *m*(*t*) which can be applied to determine the location of the rotating scatterer with respect to the transmitter and receiver in a 2D localization scenario based on Angle-of-Arrival (AoA) algorithm.), and wireless sensors to detect the asymmetry of rotation in non-destructive monitoring of rotating mechanical systems (for example the loop can be attached to a rotating shaft and whenever the shaft is not rotating around its center [$d_{L}\ne d_{R}$ in Eq. (10)] the undesired phase modulation $\psi (t)\ne 0$ can be detected as a problem occurred to the rotation of the shaft). It is a key point that in all these proposed applications, the communication process is based on a pure DAM which occupies less bandwidth compared to the Doppler modulation for a definite radius of rotation (theoretically infinite number of micro-Doppler harmonics^15,27^) and it can be demodulated even with simple envelope detectors. It should be mentioned that, although this kind of DAM for backscatter communication will be more suffered by the environment interference compared to phase (Doppler) modulation, this fact can be justified by the natural trade-off between the bandwidth and noise immunity which has always existed in telecommunication.

## Methods

### Frequency domain simulations

As it was mentioned previously, the quasi-stationary approximation can be used to solve scattering from moving scatterers. This means that the problem can be analyzed by a sequence of frequency domain simulations which correspond to the sequence of time instants along the movement. Accordingly, in each simulation the location of the scatterer should be changed such that during the full sequence of simulations, the full trajectory of the scatterer is covered. For this purpose, since the considered scatterer in this work is a wire scatterer, Method of Moments (MOM) has been selected as the faster computational approach to run the sequence of simulations with enough accuracy. The NEC2 package based on MOM has been used for doing simulation in this work. To obtain the result for a full rotation of the structure, the sequence of the simulations have been done at the desired frequency for each $1^{\circ }$ of rotation. The currents have been extracted at the middle point of the left and right radiating elements (${\tilde{I}}_{L}$ and ${\tilde{I}}_{R}$) in each structure (rectangular loop and two coupled dipoles) as it is shown in Fig. 3 by the green and red dots.

### Measurement setup

**Figure 11:**
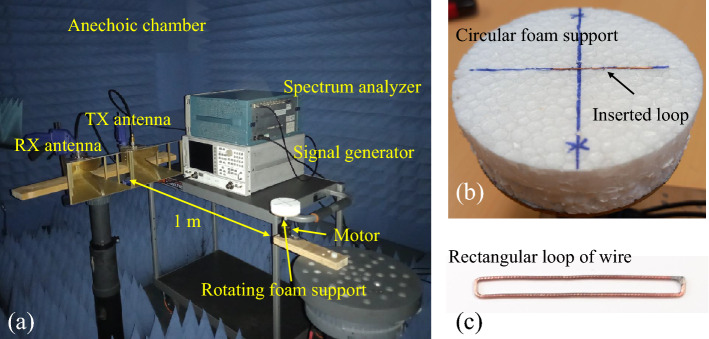
(**a**) The measurement bench in the anechoic chamber. (**b**) The circular foam support used for rotating the rectangular loop. (**c**) Fabricated rectangular loop.

The measurement bench has been organized in an electromagnetic anechoic chamber as it is shown in Fig. 11a. Two horn antennas (A.H. Systems, INC. SAS-571) with vertical linearly polarized radiation have been utilized as transmitting and receiving elements which are respectively connected to the signal generator (HP8720D in CW mode) and spectrum analyzer (Tektronix RSA3408A). The antennas are closely positioned to realize the monostatic scattering configuration. Moreover, the measurement bench and the instruments are located in the side lobe of the TX and RX antennas to reduce the undesired effect of the bench presence in the anechoic chamber. To preserve the phase variation of the backscattered signal accurately (coherent transmission and reception) the local oscillator of the signal generator and that of the spectrum analyzer have been synchronized by using a 10 MHz common reference signal. The rotation has been realized by a motor which rotates a circular foam support with a programmed speed (Fig. 11a,b). A rectangular loop has been fabricated using $250~\upmu$m copper wire (Fig. 11c), and it was inserted carefully into the slot on the foam support such that the orientation of the loop is fixed during the rotation, and it is exactly in accord with the model (Fig. 11b). The rotational frequency has been set at $f_r=40$ Hz during the measurement, while it can be set at any other frequency without affecting the results, as long as the transmitter and the receiver are coherent. A Continuous Wave (CW) signal at the frequency of $f_0=4.98$ GHz (the measured fundamental resonance frequency of the fabricated rectangular loop) has been injected into the transmitting antenna. The backscattered wave from the rotating loop has been captured by the receiving antenna, while the distance between the antennas and the rotating support is 1 m to fulfill the far-field radiation condition for both antennas and the scatterer. The received signal has been acquired by the spectrum analyzer in time (for IQ data) and frequency domain (for PSD data). The baseband demodulated I and Q data have been recorded for 160 msec acquisition time with a sampling rate of 64 kS/s, and the PSD of the signal has been measured across a 800 Hz span with a resolution of 1 Hz.

## Supplementary Information

Supplementary Information.

## Data Availability

The datasets used and/or analysed during the current study available from the corresponding author on reasonable request.
