# Supplementary material for: Directional amplitude backscatter modulation with suppressed Doppler based on rotating resonant loop
Source: Sci Rep. 2022 Dec 20;12:22032. doi: 10.1038/s41598-022-26609-w (PMC9767923; doi:10.1038/s41598-022-26609-w)
Supplement: Supplementary file 1 — Supplementary Information. [file 41598_2022_26609_MOESM1_ESM.pdf]

Supplementary Part

Supplementary A

For the given V_L and V_R, the circuit model shown in Figure 1 can be solved using the superposition theorem to obtain the flowing currents at the left (I_L) and right (I_L) sides of the transmission line. The problem can be decomposed to the even and odd excitation, respectively shown in Figure 2 (a) and (b), which yield the solution for even and odd resonant modes of the loop structure. Afterward, the general solution is provided by the summation of all even and odd solutions (modes).

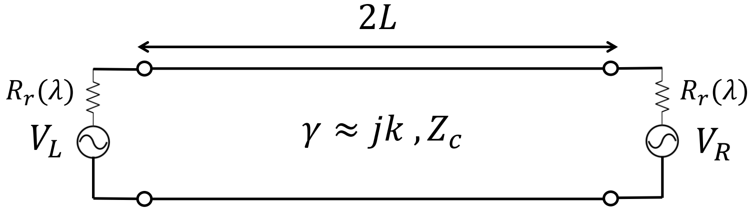

Figure 1: Proposed circuit model for rectangular loop excited by vertically polarized plane wave.

Due to the symmetry of the even and odd excitation, the center point of the transmission line can be replaced by an open-circuit in even modes and a short-circuit point in odd modes. Accordingly, the flowing current through the short sides of the loop in each mode (equal and in-phase currents I_e for even modes and equal but oppositely directed currents for odd modes I_o) can be calculated as

$$I_e = \frac{V_e}{Z_e} = \frac{V_e}{R_r(\lambda) - jZ_c \cot(kL)} \tag{1}$$

$$I_o = \frac{V_o}{Z_o} = \frac{V_o}{R_r(\lambda) + jZ_c \tan(kL)} \tag{2}$$

where Z_e and Z_o are respectively the impedance seen by the voltage sources in even and odd mode, and the general currents can be written as $I_L = I_e + I_o$ and $I_R = I_e - I_o$.

The resonance frequencies of the circuit will be defined based on the imaginary part of the Z_e and Z_o. The resonance occurs when the imaginary part approaches zero and magnitude of the current is maximized. So, the even and odd resonance frequencies are obtained by

$$\text{Even modes} \rightarrow \cot(kL) = 0 \rightarrow kL = \frac{(2n+1)\pi}{2} \quad n = 0, 1, 2, ... \rightarrow \lambda_r^{even} = \frac{4L}{(2n+1)} \tag{3}$$

$$\text{Odd modes} \rightarrow \tan(kL) = 0 \rightarrow kL = n\pi \quad n = 1, 2, ... \rightarrow \lambda_r^{odd} = \frac{2L}{n} \tag{4}$$

According to (3) and (4) the fundamental resonant mode of the structure occurs at $L = \lambda/4$ (dominant even mode with $n = 0$) where $Z_o = \infty$ and consequently $I_o = 0$. Therefore, at the fundamental mode the currents are

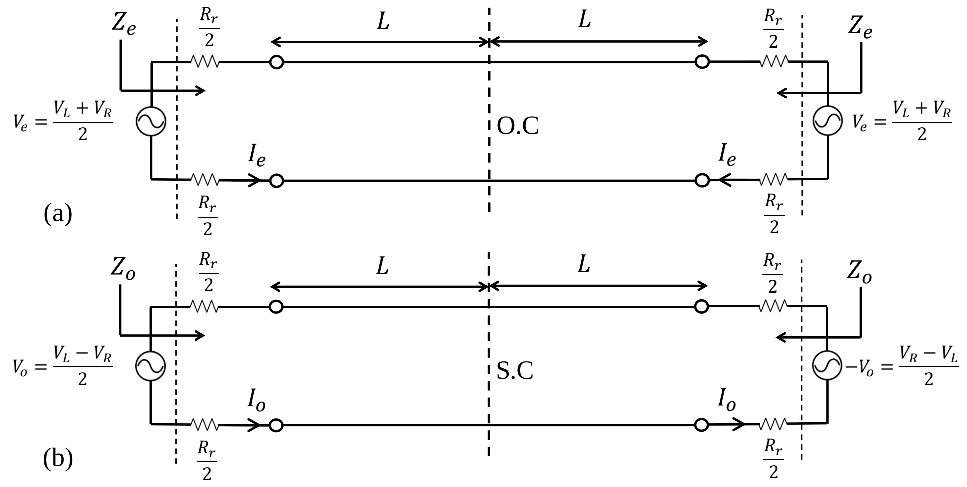

Figure 2: (a) Equivalent circuit for even mode excitation. (b) Equivalent circuit for odd mode excitation.

derived as

$$I_L = I_R = I_e = \frac{V_L + V_R}{2R_r(\lambda = 4L)} \tag{5}$$

which shows that in the fundamental mode the two reradiating current components are exactly identical, and they can be supposed as $I_L = I_R = I_0$.

Supplementary B

The vector effective length of the loop associated to its fundamental mode can be derived using the method presented in [1] based on reciprocity theorem. Two reciprocal configuration consist of the rectangular loop and a circular electrically small loop with radius of $b \ll \lambda$ are shown in Figure 3. In the first configuration [Figure 3 (a)], two radiating current elements are considered on short sides of the rectangular loop and the open-circuited circular loop is assumed at a far-field point $P : (r, \theta, \phi)$ while it is normal to the $\hat{\phi}$ direction. The radiated magnetic field form the two current elements (right and left side element) has only non-zero ϕ-component which can be expressed as

$$H_\phi^L = \frac{j2hkI_0}{4\pi r_L} \sin\theta \, e^{-jkr_L} \simeq \frac{j2hkI_0 e^{-jkr}}{4\pi r} \sin\theta \, e^{jkd_L \sin\theta\cos\phi} \tag{6}$$

$$H_\phi^R = \frac{j2hkI_0}{4\pi r_R} \sin\theta \, e^{-jkr_R} \simeq \frac{j2hkI_0 e^{-jkr}}{4\pi r} \sin\theta \, e^{-jkd_R \sin\theta\cos\phi} \tag{7}$$

where r_L and r_R are respectively associated to the position of the left and right side currents which are approximated at the far-field by $r_R \simeq r_L \simeq r$ for the amplitude, and $r_L \simeq r - d_L \sin\theta\cos\phi$, $r_R \simeq r + d_R \sin\theta\cos\phi$ for the phase. The total radiated magnetic field H_ϕ is obtained by complex summation of (6) and (7) which can be written as

$$H_\phi = H_\phi^L + H_\phi^R = \frac{j4hkI_0 e^{-jkr}}{4\pi r} \sin\theta \, e^{-jk\frac{(d_R - d_L)}{2}\sin\theta\cos\phi} \cos(kL\sin\theta\cos\phi) \tag{8}$$

where $L = \frac{(d_L + d_R)}{2}$ is half of the loop length. Based on the Faraday's law of induction, the induced open-circuit voltage $V_{\alpha\beta}$ at the port of the small circular loop can be obtained as

$$V_{\alpha\beta} = -j\omega\mu_0\pi b^2 H_\phi \tag{9}$$

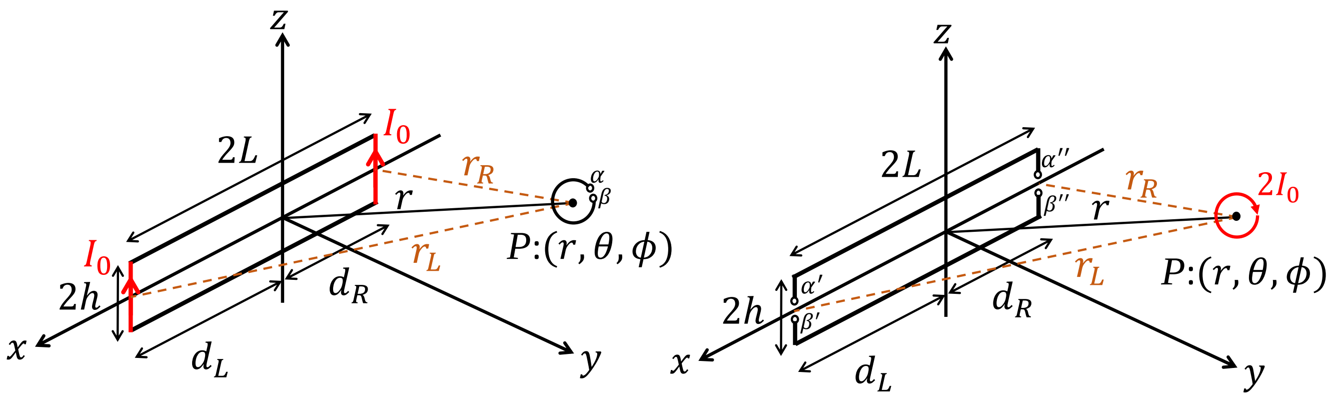

Figure 3: Two proposed reciprocal configuration used to obtain effective length of the loop in its fundamental mode. (a) First configuration; excited rectangular loop and open-circuited small loop. (b) Second configuration; excited small loop and open-circuited rectangular loop

In the second configuration (reciprocal configuration) as it is shown in Figure 3 (b), the current elements and open-circuit ports are replaced for rectangular loop and small loop. Accordingly, the uniform current of $2I_0$ is assumed on the circular loop and two open-circuit ports ($\alpha'\beta'$ and $\alpha''\beta''$) are considered on two short sides of the rectangular loop. The radiated electric field from electrically small circular loop with uniform current of $2I_0$, when it is positioned normal to the $\hat{\phi}$ direction, has only a non-zero θ-component written as [1]

$$E_\theta^{loop}(\vec{r}) = 2\eta I_0 (kb)^2 \frac{e^{-jkr}}{4r} \tag{10}$$

Based on the reciprocity theorem [2], the two equal and in-phase open-circuit voltage induced on the ports of the rectangular loop ($V_{\alpha'\beta'} = V_{\alpha''\beta''}$) will be identical to that has been obtained in (9). It should be noted that the assumption of $V_{\alpha'\beta'} = V_{\alpha''\beta''}$ is imposed by the excited fundamental even mode in the loop. Finally, the ratio between the open-circuit voltages ($V_{\alpha'\beta'}$ and $V_{\alpha''\beta''}$) and $E_\theta^{loop}(\vec{r})$ at the position of the ports ($r_{\alpha'\beta'} = r_L$ and $r_{\alpha''\beta''} = r_R$) will yield the θ-component of vector effective length associated to each current elements on short sides of the loop as

$$l_{e\theta}^L(\theta,\phi) = \frac{V_{\alpha'\beta'}}{E_\theta^{loop}(r_{\alpha'\beta'})} = 2h \sin\theta \cos(kL \sin\theta\cos\phi)\, e^{-jkL\sin\theta\cos\phi} \tag{11}$$

$$l_{e\theta}^R(\theta,\phi) = \frac{V_{\alpha''\beta''}}{E_\theta^{loop}(r_{\alpha''\beta''})} = 2h \sin\theta \cos(kL \sin\theta\cos\phi)\, e^{jkL\sin\theta\cos\phi} \tag{12}$$

The above procedure can be repeated when the small circular loop is oriented normal to the $\hat{\theta}$ direction to obtain the ϕ-component of the vector effective length of the rectangular loop. However, as the θ-component of the radiated magnetic field from rectangular loop is zero, consequently, the induced open-circuit voltage on the small loop ($V_{\alpha\beta}$) and the ϕ-component of the vector effective length will be zero too. Therefore, (11) and (12) describe the complete expression for vector effective length of the loop in its fundamental mode which can be used to calculate V_L and V_r in terms of the incident electric field, and finally to obtain the currents $I_L = I_R = I_0$ using (5).

References

[1] A. Wunsch, "The vector effective length of slot antennas," *IEEE Transactions on Antennas and Propagation*, vol. 39, no. 5, pp. 705–709, 1991.

[2] R. F. Harrington, *Time-Harmonic Electromagnetic Fields*. Wiley-IEEE Press, 2001, ch. 2.
